# A Novel Germline Heterozygous *BCL11B* Variant Causing Severe Atopic Disease and Immune Dysregulation

**DOI:** 10.3389/fimmu.2021.788278

**Published:** 2021-11-23

**Authors:** Henry Y. Lu, Robert Sertori, Alejandra V. Contreras, Mark Hamer, Melina Messing, Kate L. Del Bel, Elena Lopez-Rangel, Edmond S. Chan, Wingfield Rehmus, Joshua D. Milner, Kelly M. McNagny, Anna Lehman, David L. Wiest, Stuart E. Turvey

**Affiliations:** ^1^ Department of Pediatrics, British Columbia Children’s Hospital, The University of British Columbia, Vancouver, BC, Canada; ^2^ Experimental Medicine Program, Faculty of Medicine, The University of British Columbia, Vancouver, BC, Canada; ^3^ Blood Cell Development and Function Program, Fox Chase Cancer Center, Philadelphia, PA, United States; ^4^ Biomedical Research Centre, The University of British Columbia, Vancouver, BC, Canada; ^5^ Department of Pediatrics, Columbia University Irving Medical Center, New York, NY, United States; ^6^ Department of Medical Genetics, The University of British Columbia, Vancouver, BC, Canada

**Keywords:** primary atopic disorders, inborn errors of immunity, primary immunodeficiencies, hyper IgE, *BCL11B*

## Abstract

B-cell lymphoma/leukemia 11B (BCL11B) is a C_2_H_2_ zinc finger transcription factor that is critically important for regulating the development and function of a variety of systems including the central nervous system, the skin, and the immune system. Germline heterozygous variants are associated with a spectrum of clinical disorders, including severe combined immunodeficiency as well as neurological, craniofacial, and dermal defects. Of these individuals, ~50% present with severe allergic disease. Here, we report the detailed clinical and laboratory workup of one of the most severe BCL11B-dependent atopic cases to date. Leveraging a zebrafish model, we were able to confirm a strong T-cell defect in the patient. Based on these data, we classify germline BCL11B-dependent atopic disease as a novel primary atopic disorder.

## Introduction

Primary atopic disorders (PADs) are a group of monogenic disorders that present with dysregulated allergic effector responses (1). Major clinical features include severe atopic dermatitis, food allergies, allergic asthma, urticaria, eosinophilia, and elevated IgE. Many PADs also have comorbid immunodeficiency and immune dysregulation such as systemic lupus erythematosus and autoimmune vasculitis as is the case in some patients with dominant-negative *STAT3* variants (1). Due to the heterogeneous presentation of PADs, it can often be difficult to differentiate monogenic from polygenic etiologies. However, the increased clinical implementation of diagnostic next-generation sequencing has led to increased identification of PAD patients.

*B-cell lymphoma/leukemia 11B* (*BCL11B*) is a C_2_H_2_ zinc finger (ZF) transcription factor protein that is broadly expressed and is important in regulating the development of various tissues, including the central nervous system, T cells, skin, and teeth (2). It serves as both a transcriptional activator and repressor and binds GC-rich response elements (2). Germline heterozygous variants in *BCL11B* have been identified in ~17 patients thus far (extracted from publications written in English) and are associated with a range of clinical phenotypes. This includes severe combined immunodeficiency (SCID) with neurological, craniofacial, and dermal abnormalities, coronal suture synostosis, short stature, and intellectual disability, speech delay, dysmorphic facies, dental abnormalities, and T-cell and innate lymphoid cell defects (3–5). Notably, eight of 17 (~47%) of these patients presented with atopic disease, including asthma, eosinophilia, food allergies, and eczema (4). Here, we present the case of a female patient with severe atopic disease, neurodevelopmental abnormalities, and immune dysregulation, who carries a novel germline heterozygous variant in *BCL11B.* We propose heritable *BCL11B*-related atopic disease as a novel PAD.

## Materials and Methods

### Study Participants and Consent

All study participants and/or their parents/guardians provided written informed consent to participate. All individuals also provided consent to be published. Research study protocols were approved by The University of British Columbia Clinical Research Ethics Board.

### *BCL11B* Variant Prioritization

The potentially pathogenic NM_138576:c.2487G>A;NP_612808:p.Cys826Tyr *BCL11B* variant was selected because (i) germline *BCL11B* variants have been associated with both neurodevelopmental disorders as well as atopic disease; (ii) it is absent in population databases (e.g., gnomAD); and (iii) it is predicted to be pathogenic by a variety of *in silico* pathogenicity prediction tools. Notably, VarCards, which incorporates 23 pathogenicity prediction algorithms, including SIFT, Polyphen-2, MutationTaster, MutationAssessor, FATHMM, and more, predicts the p.Cys826Tyr BCL11B variant to be pathogenic as determined by 21/23 algorithms. Furthermore, this variant has a combined annotation-dependent depletion (CADD) score of 23, which is far above its mutation significance cutoff (MSC) of 3.313.

### Zebrafish Modeling

To investigate the impact of the p.Cys826Tyr BCL11B variant on T-cell development, wild-type (WT) or p.Cys826Tyr human BCL11B was ectopically expressed in one-cell-stage zebrafish embryos for which the conserved zebrafish BCL11B orthologue *bcl11ba* had been knocked down, as previously described (3). Briefly, *bcl11ba* morpholino oligonucleotides were injected into one-cell embryos to block translation and splicing of zebrafish *bcl11ba* along with either WT or p.Cys826Tyr *BCL11B* in a heat-inducible pSGH2 vector. 30 h post-fertilization, embryos were heated at 37°C for 1 h to induce WT or p.Cys826Tyr BCL11B expression. To visualize the development of T-cell progenitors, 5 days post-fertilization, zebrafish embryos were subjected to whole-mount *in situ* hybridization (WISH) with a *lck* probe.

## Results

### Patient Clinical Presentation of Disease

**Figure 1:**
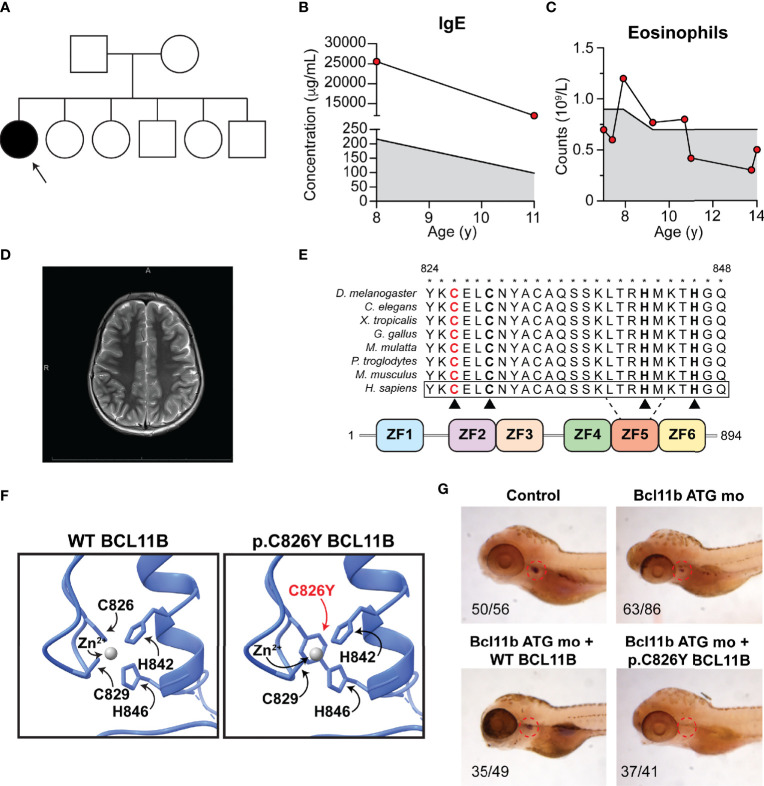
Patient phenotype and functional validation of a novel BCL11B variant. **(A)** Family pedigree. Arrow, proband. **(B)** Patient IgE at ages 8 and 11. **(C)** Patient eosinophil counts from ages 7 to 14. **(B, C)** Shaded region, reference range. **(D)** Patient brain magnetic resonance imaging. **(E)** Schematic of BCL11B protein. Red, substitution location. Asterisks, conservation. Black triangles, zinc coordinating residues. ZF, zinc finger. **(F)** Homology-based modeling of affected region. **(G)** Rescue of T-cell development visualized by *lck* probe post-*bcl11ba* knockdown with Bcl11b ATG mo and heat-inducible reexpression of WT (bottom left) or p.Cys826Tyr BCL11B (bottom right). Indicated are the number of individuals with observed phenotype.

The patient is a 14-year-old Canadian girl born to non-consanguineous parents (**Figure 1A**) who presented with microcephaly, mild intellectual disability, severe and broad food allergies (**Table 1**), atopic dermatitis, asthma, elevated IgE (**Figure 1B**), eosinophilia (**Figure 1C**), alopecia totalis, brittle nails, prurigo nodularis, and antinuclear antibodies. In particular, the food allergy diagnosis was based on three converging lines of evidence: (1) history consistent with IgE-mediated immediate hypersensitivity reactions (i.e., urticaria, lip swelling, vomiting, diarrhea) following exposure to egg, peanut, hazelnut, sesame, sunflower seed, tuna, and liquid cow’s milk); (2) positive epicutaneous skin prick testing to cow’s milk, salmon, peanuts, treenuts, and seeds, as well as multiple environmental allergens, including tree and grass pollen, cat, dog, and dust mite; and (3) the presence of serum-specific IgE. A brain magnetic resonance imaging (MRI) scan was normal and did not reveal any evidence of corpus callosum agenesis as was reported in a previous case (**Figure 1D**) **(** 3). Family history was unremarkable; the patient’s five siblings are healthy.

**Table 1:** Allergen-specific IgE.

Allergen IgE (>0.35 kU/L = positive)	
Almond (17.70)	Walnut (71.80)
Cashew nut (52.30)	Egg white (99.50)
Hazelnut (32.10)	Egg yolk (76.50)
Peanut (85.50)	Tuna (19.40)
Pecan/Hick nut (18.00)	Salmon (73.50)
Pine nut (2.65)	Halibut (42.20)
Pistachio (53.30)	Codfish (41.00)
Cow’s milk (20.8)	Sesame seed (97)
Sunflower seed (56.6)	

Tabulation of a panel of allergen-specific IgE levels measured in the patient.

### Genetic Workup

Given the unique combination of clinical features, the patient underwent trio whole exome sequencing, which revealed a novel *de novo* missense NM_138576:c.2487G>A;NP_612808:p.Cys826Tyr *BCL11B* variant (**Figure 1E**). This variant has never been reported in any population databases and was predicted to be pathogenic by a large range of *in silico* pathogenicity prediction tools, including 21/23 algorithms used by VarCards (6). Notably, this variant had a CADD score of 23, where the suggested CADD cutoff for *BCL11B* according to MSC is 3.313 (7). Cys826 in the fifth zinc finger of the protein was highly conserved through evolution (**Figure 1E**). Homology-based structural modeling using the crystal structure of the related BCL11A transcription factor (8) revealed that the p.Cys826Tyr substitution likely perturbs the coordination of zinc ions, which is important for protein-protein interactions and the formation of protein complexes (2) (**Figure 1F**).

### Detailed Clinical Laboratory Workup

Since BCL11B is critically important for regulating T-cell development and function (2), we carried out detailed immunophenotyping of the patient. Surprisingly, we discovered that the patient had a modest B-cell developmental defect (elevated naïve B cells and decreased memory B cells) (**Table 2**). However, patient T-cell numbers were generally unremarkable. T-cell receptor excision circles (TRECs) were within the reference range, thymic output was normal, and CD4^+^ and CD8^+^ T-cell subsets (naïve, terminally differentiated, central memory, effector memory, Th17, Treg, etc.) were all comparable with controls (**Table 2**). Furthermore, mitogen proliferation responses were normal.

**Table 2:** Patient laboratory values.

	Patient (11 years old)		Reference range (10–16 years old)	
	Abs. # (×10^9^/L)	%	Abs # (×10^9^/L)	%
B cells	0.294	11	0.120–0.740	7–24
Memory B cells	0.022^*^	7.5^*^	0.050–0.200	13.3–47.9
Naïve B cells	0.283	96.1^*^	0.120–0.430	51.3–82.5
Non-switched memory B cells	0.019^*^	6.5	0.020–0.070	4.6–18.2
Class-switched memory B cells	0.003^*^	0.9^*^	0.030–0.110	8.7–25.6
IgM+ memory B cells	0.010	3.3	0.002–0.013	0.5–7.0
Transitional B cells	0.044	15.0^*^	0.010–0.060	1.4–13.0
Activated CD21lo CD38lo B cells	0.008	2.8	0.004–0.037	1.0–11.0
Immature CD21lo B cells	0.023	7.7	0.010–0.050	2.9–13.2
Plasmablasts	0.003	1.0	0.000–0.020	0.6–6.5
T cells	2.090	80.8	0.850–3.200	52–90
Recent thymic emigrants	0.7	57	0.2–1.5	31–81
T helper cells	1.160	45.0	0.400–2.100	20–65
Naive	0.790	68	0.200–1.700	37–97
Terminally differentiated	0.002	0	0.000–0.051	0–6
Central memory	0.339	29	0.120–0.740	13–76
Effector memory	0.029	2	0.005–0.210	1–25
Th17	–	0.676	–	0.31–1.80
Treg	0.096	8.3	0.033–0.190	4.0–20.0
Cytotoxic T cells	0.720	27.9	0.300–1.300	14–40
Naive	0.587	82	0.078–0.640	20–95
Terminally differentiated	0.033^*^	5^*^	0.035–0.420	9–65
Central memory	0.069	10	0.002–0.086	0–18
Effector memory	0.031	4	0.016–0.810	4–100
Double-negative T cells	0.170	6.7	–	–
TCRαβ+	1.66	6.5	0.70–2.80	39–92
TCRγδ+	0.12	4.7	0.04–0.54	2.0–17.0
CD4/CD8 ratio	–	1.61	–	0.9–3.4
NKT cells	0.260	10.0	0.016–0.350	1–15
iNKT cells	0.000100	*0.005	0.000100–0.000624	0.008–0.374
TREC (copy #/3 µl)	327		147–1,330	

Tabulation of patient immune cell proportions compared with an age-specific reference range. Abnormal values are marked with asterisks (*) and are set in bold. #, number.

### Zebrafish Modeling of the p.Cys826Tyr BCL11B Variant

To evaluate whether the p.Cys826Tyr BCL11B variant is disease causing, we assessed the impact of this variant on T-cell development in zebrafish as previously described (3). Briefly, we knocked down the BCL11B zebrafish orthologue *bcl11ba* by injecting *bcl11ba* antisense morpholino oligonucleotides into zebrafish embryos and then ectopically expressed WT BCL11B or p.Cys826Tyr BCL11B to investigate whether T-cell development is rescued. Confirming the pathogenicity of the p.Cys826Tyr BCL11B variant, the mutant variant was unable to rescue T-cell development as zebrafish ectopically expressing the mutant variant had no detectable thymocytes as measured by a *lck* probe on WISH (**Figure 1G**).

## Discussion

Here, we report one of the most severe atopic cases of BCL11B deficiency to date and the first known Canadian patient. To our knowledge, this is also the first patient with germline *BCL11B* variants associated with immune dysregulation/autoimmunity. Based on the clinical features of this patient and the eight previous individuals with atopic features (summarized and contrasted in **Table 3**), we propose to classify germline BCL11B-related atopic disease as a novel PAD. This case further emphasizes the fact that PADs can have heterogeneous clinical presentations, which can often appear to be benign especially relative to inborn errors of immunity. Based on our data and ACMG/AMP guidelines, the p.Cys826Tyr BCL11B variant can now be classified as pathogenic as it satisfies PS2, PS3, PM2, and PP3 criteria.

**Table 3:** Atopy associated with germline *BCL11B* variants.

BCL11B variant	46,XY,t(4;14)(p15;q32.1)	p.Cys81Leufs*76	p.Tyr455*	p.Glu499*	p.Arg518Alafs*45	p.Asn807Lys	p.Cys826Tyr	p.Ala891Profs*67	%
Atopic dermatitis	–	–	+	ND	–	–	+	–	29
Asthma	–	+	+	ND	+	–	+	+	71
(Food) allergies	+	–	+	ND	–	–	+	–	43
lgE	ND	ND	ND	ND	ND	ND	+	ND	
Eosinophilia	+	+	ND	–	+	+	+	–	71

Tabulation of major atopic features observed in patients found to carry germline pathogenic BCL11B variants. Frequencies of each phenotype are indicated in the last column. These data were extracted only from manuscripts written in English. ND, no data-, not reported in patient; +, reported in patient.

Although our in-depth immunophenotyping of the patient revealed intact T-cell numbers, our zebrafish model demonstrated that the p.Cys826Tyr BCL11B variant was unable to rescue T-cell development. Despite this mismatch, patient T cells are clearly defective as the patient presented with alopecia and allergic disease, both of which are T-cell dependent (9, 10). In the future, it would be important to study how immune deficiency arose in this patient despite intact thymic development (normal TRECs and recent thymic emigrants), in contrast to the ablated T-cell development observed in zebrafish. For example, since BCL11B is essential for multiple T-cell developmental checkpoints in the thymus (11), one possibility is dysregulated thymic architecture. This could be investigated by performing patient-derived CD34^+^ cell xenotransplants or leveraging artificial thymic organoid systems (12).

Given the modestly impaired B-cell differentiation, a part of the immunodeficiency could be derived from impaired T helper function (11). It is possible that the p.Cys826Tyr BCL11B variant alters the binding affinity of WT BCL11B to its targets or causes protein complexes to bind to new targets, leading to a predisposition to developing allergic disease. For example, p.Cys826Tyr BCL11B could continuously induce GATA3 expression without silencing IL-4 production (13). However, since the C-terminal ZFs are thought to regulate a specific subset of BCL11B activities through as-yet-unidentified binding partners (2), further investigation is needed. This is complicated by the fact that BCL11B target genes in mature effector T cells are not well understood nor is it clear how or whether BCL11B has a role in promoting or restricting the plasticity of T helper subsets other than T_H_17 and Tregs (2). Future work should also focus on clarifying why atopic disease arises in some individuals with germline *BCL11B* variants but not others and how specific variants lead to atopy.

## Data Availability Statement

The raw data supporting the conclusions of this article will be made available by the authors, without undue reservation.

## Ethics Statement

The studies involving human participants were reviewed and approved by the Clinical Research Ethics Board, The University of British Columbia. Written informed consent to participate in this study was provided by the participants’ legal guardian/next of kin. The animal study was reviewed and approved by the Institutional Animal Care and Use Committee, Fox Chase Cancer Center.

## Author Contributions

HL performed immunophenotyping, obtained clinical data, carried out the multiple sequence alignment, *in silico* pathogenicity prediction, and protein modeling, wrote the manuscript, and made the figures. RS, AC, and DW modeled the *BCL11B* variant in zebrafish. MH, MM, KD, and KM helped with writing and editing the manuscript. EL-R, EC, WR, JM, and AL collected clinical data and helped write and edit the manuscript. ST supervised all the research, provided funding, contributed to experimental design and analysis, and helped write and edit the manuscript. All authors contributed to the article and approved the submitted version.

## Funding

This work was supported by grants from the Canadian Institutes of Health Research (ST), Genome British Columbia (SIP007) (ST), Rare Disease Foundation (2174) (HL and ST), and BC Children’s Hospital Foundation (CAUSES Study, HL, ST). ST holds a Tier 1 Canada Research Chair in Pediatric Precision Health and the Aubrey J. Tingle Professor of Pediatric Immunology. HL is supported by a Canadian Institutes of Health Research Frederick Banting and Charles Best Canada Graduate Scholarship (CGS-D), Killam Doctoral Scholarship, University of British Columbia Four Year Doctoral Fellowship, and a BC Children’s Hospital Research Institute Graduate Studentship. DW was supported by the National Institutes of Health grants P30CA006927 and P01AI138962.

## Conflict of Interest

The authors declare that the research was conducted in the absence of any commercial or financial relationships that could be construed as a potential conflict of interest.

## Publisher’s Note

All claims expressed in this article are solely those of the authors and do not necessarily represent those of their affiliated organizations, or those of the publisher, the editors and the reviewers. Any product that may be evaluated in this article, or claim that may be made by its manufacturer, is not guaranteed or endorsed by the publisher.
